# Multifunctional PMMA@Fe_3_O_4_@DR Magnetic Materials for Efficient Adsorption of Dyes

**DOI:** 10.3390/ma10111239

**Published:** 2017-10-27

**Authors:** Bing Yu, Liang He, Yifan Wang, Hailin Cong

**Affiliations:** 1 Institute of Biomedical Materials and Engineering, College of Chemistry and Chemical Engineering, Qingdao University, Qingdao 266071, China; yubingqdu@yahoo.com (B.Y.); lianghe169@163.com (L.H.); wangyifan@qdu.edu.cn (Y.W.); 2 Laboratory for New Fiber Materials and Modern Textile, Growing Base for State Key Laboratory, College of Materials Science and Engineering, Qingdao University, Qingdao 266071, China

**Keywords:** poly (methylmethacrylate) (PMMA), magnetic, diazo-resin (DR), adsorption, dye

## Abstract

Magnetic porous microspheres are widely used in modern wastewater treatment technology due to their simple and quick dye adsorption and separation functions. In this article, we prepared porous polymethylmethacrylate (PMMA) microspheres by the seed-swelling method, followed by in situ formation of iron oxide (Fe_3_O_4_) nanoparticles within the pore. Then, we used diazo-resin (DR) to encapsulate the porous magnetic microspheres and achieve PMMA@Fe_3_O_4_@DR magnetic material. We studied the different properties of magnetic microspheres by different dye adsorption experiments before and after the encapsulation and demonstrated that the PMMA@Fe_3_O_4_@DR microspheres can be successfully used as a reusable absorbent for fast and easy removal of anionic and aromatic dyes from wastewater and can maintain excellent magnetic and adsorption properties in harsh environments.

## 1. Introduction

Magnetic nanoparticles are currently being actively investigated because of their extensive applications, such as in magnetic imaging [1], biological separation [2], drug release [3], and water purification [4]. Textiles, clothing, printing, and dyeing processes release a lot of dyes, which are considered toxic and may cause cancer and other diseases [5,6,7,8,9,10]. However, most of the dyes in water are very difficult to remove. The removal of dyes from the environment has been widely studied and numerous methods have been carried out, such as adsorption, membrane filtration, oxidation, photocatalyzation, etc. [11,12,13,14,15]. Physical adsorption is generally recognized as a simple and efficient technique. Magnetic porous microspheres have many applications in modern wastewater treatment technology due to their simple and quick dye adsorption and separation functions [16,17,18,19,20,21,22,23]. Moreover, it is a very useful way of refining the purification of hard materials, such as protein [24].

Polymethylmethacrylate (PMMA) resin is a non-toxic and environmentally-friendly material, which can be used in the production of tableware, sanitary ware, etc. It has good chemical stability and weather resistance [25]. However, few people have synthesized PMMA porous microspheres. We successfully synthesized PMMA porous microspheres with good monodispersity and regularity by the seed-swelling method [26], followed by the in-situ formation of iron oxide nanoparticles in the tunnel and applied them to wastewater treatment.

Acid dyes are water-soluble dyes with acidic groups in the acid medium for dyeing. Most acid dyes contain sodium sulfonate salts that are soluble in water, which makes them difficult to remove from aqueous solutions [27,28,29,30]. The acidic character makes them easily corrode magnetic materials, so the encapsulation of magnetic materials is very necessary. The incorporation of the photo-curable crosslinking oligomer, diazo-resin (DR) 4-diazodiphenylamine attached to PMMA porous microspheres by ionically-bonded layer-by-layer films is a promising approach for increasing magnetic material stability [31]. DR is a cationic polymer that allows for the conversion of the ionic bond to the covalent bond with various negatively-charged functional groups, by the decomposition of its diazonium group under ultraviolet (UV) irradiation [32]. The diazo group is converted to a secondary amino group, so the original surface with a negative charge changes to the positive. Hence, the magnetic microspheres are encapsulated and exhibit more excellent stability in harsh environments.

In the present work, firstly, the monodispersed PMMA seeds were synthesized by dispersion polymerization. Secondly, the PMMA porous microspheres were synthesized by seed swelling. Then, in situ formation of iron oxide nanoparticles in the tunnel was carried out and applied to adsorb dyes. Finally, DR was used to encapsulate PMMA porous magnetic microspheres. We demonstrated that with DR encapsulation, the PMMA@Fe_3_O_4_@DR magnetic microspheres can maintain excellent magnetic and adsorption properties in harsh environments. Compared with PMMA@Fe_3_O_4_ magnetic microspheres, the DR encapsulated counterparts exhibit better stability.

## 2. Experiment

### 2.1. Materials

Diazo resin (Mn = 2500) [33], methyl methacrylate (MMA, 99.5%, Qingdao Renhe Xing Experimental Technology Co., Ltd., Qingdao, China) were purchased from Qingdao Renhe Xing Experimental Technology Co., Ltd., and were distilled under vacuum before use. Ethylene glycol dimethacrylate (EGDMA, Aladdin Biochemical Technology Co., Ltd., Shanghai, China) was used as crosslinker. Polyvinyl pyrrolidone (PVP K-30, 99%) was purchased from Qingdao Renhe Xing Experimental Technology Co., Ltd., Sodium dodecyl sulfate (SDS), 2,2-azobis(isobutyronitrile) (AIBN), benzoyl peroxide (BPO) and dibutyl phthalate (DBP) (Tianjin Chemical Company, Tianjin, China) were purchased from Tianjin Chemical Company. AIBN and BPO were purified by recrystallization. All the chemicals were used as received unless noted elsewhere.

### 2.2. Preparation of Seed Particles by Dispersion Polymerization

The monodisperse PMMA seed particles were fabricated by dispersion polymerization methods [34], in a methanol medium with PVP K-30 as a stabilizer and AIBN as an initiator. In a typical experiment, 5.4 g of MMA, 34 g of methanol, 14 g of H_2_O, 1 g of AIBN and 1 g of PVP were added to a three-necked round-bottom flask, which was equipped with a mechanical stirrer and ultrasonic mixing, followed by stirring into a homogeneous phase and purging with nitrogen for 30 min. After the temperature was slowly elevated to 65 °C, the stirring rate was maintained at 40 rpm for 24 h. The PMMA spheres were obtained by centrifugation and repeatedly washed with ethanol to remove residual MMA and PVP. Thereafter, the beads were dried under a vacuum at ambient temperature.

### 2.3. Preparation of Porous Microspheres by a Two-Step Seed Emulsion Polymerization

We prepared the porous microspheres by a two-step seed emulsion polymerization [35]. Firstly, the seed particles (0.26 g) were ultrasonically redispersed in a 10 mL deionized water solution, followed by the addition of 1.2 mL DBP, toluene (1.2 mL) and 0.375% SDS water solution (20 mL) into the beaker. It was redispersed by sonication for 30 min, and then the mixture was stirred for 24 h at 30 °C. Secondly, the mixture of MMA (0.6 mL), EGDMA (0.6 mL), (15 mL), and BPO (0.1 g) were poured into the beaker, which had been emulsified with 100 mL of a 0.25% SDS solution by sonication for 30 min. The swelling stage of the monomer was held for another 24 h at 30 °C. Finally, 3.5 mL of a 10 wt % PVA aqueous solution was added to the reactor. The polymerization was carried out at 70 °C for 24 h. The product was obtained by repeated centrifugation, followed by washing three times with ethanol and deionized water. The product was dissolved in hot tetrahydrofuran to remove the porogen and the linear polymer. The resulting microspheres were washed with ethanol and deionized water, then dried under vacuum.

### 2.4. Hydrolysis of PMMA Porous Microspheres

The PMMA porous microspheres (0.1 g) were dispersed in ethanol for 24 h and centrifuged to remove excess ethanol. The porous spheres were swelled and added to the aqueous 15 wt % NaOH solution, then the mixture was stirred for 5 h at 45 °C, followed by washing three times with deionized water so that pH = 7. 

### 2.5. Preparation of Porous Magnetic PMMA Microspheres 

The PMMA-COOH microspheres (0.1 g) were transferred into a 250 mL three -necked round-bottom flask, and then 30 mL of a 0.043 g FeCl_2_·4H_2_O, 0.118 g FeCl_3_·6H_2_O solution were added to the flask and stirred for 5 h under a nitrogen atmosphere. After that, the microspheres were separated by centrifugation and washed with deionized water until no Fe^2+^ and Fe^3+^. The microspheres were ultrasonically redispersed in 30 mL deionized water solution. The temperature was raised to 80 °C, 5 mL aqueous ammonia was added and carried out for 0.5 h. The product was centrifuged through deionized water, then dried under vacuum.

### 2.6. Preparation of PMMA@Fe_3_O_4_@DR Magnetic Materials

The preparation of photosensitive DR was according to previous work [34]. The magnetic PMMA microspheres (0.1 g) were transferred into a 15 mL black glass bottle, and then 10 mL DR (50 g/L) solution was mixed, followed by shaking for 24 h at a low temperature and in a dark environment. The microspheres were separated by centrifugation, then dried under vacuum. Finally, the product was irradiated for 30 min under a UV lamp, and PMMA@Fe_3_O_4_@DR magnetic materials was obtained.

### 2.7. Adsorption of Organic Dye

Five kinds of adsorbates were used in the adsorption experiments (see Figure 6d). Typically, 50 mg of PMMA@Fe_3_O_4_-COOH and PMMA@Fe_3_O_4_@DR adsorbent was mixed with 10 mL of solution at the desired dyes concentrations, pH, 25 °C. After the agitation at a rate of 100 rpm for 30 min, the solution was centrifuged and a lot of the liquid was taken to be analyzed by UV-vis absorption spectroscopy by monitoring the absorbance changes at a wavelength of maximum absorbance after being diluted twice. The effect of pH on the amount of dyes removal was analyzed in the pH range from 2 to 10. The solution pH was adjusted by using of 0.1 M HCl solution and 0.1 M NaOH solution.

### 2.8. Characterization

The morphology and size of the obtained microspheres were characterized by using scanning electron microscopy (SEM, JEOL JSM-6309LV, Beijing, China). Magnetic measurements of the PMMA@Fe_3_O_4_@DR magnetic microspheres were carried out on an alternate gradient magnetometer (Micro MagTM 2900, Shanghai Yi Hong Scientific Instrument Co., Ltd., Shanghai, China) at room temperature. The magnetic field was created by a superconducting solenoid in the persistent mode, and the hysteresis loop was recorded in the field up to 4.5 kOe. Thermogravimetric analysis (TGA) was carried on a Perkin-Elmer (PE) TGA-7 instrument (Shanghai Perkin Elmer Enterprise Management Co., Ltd., Shanghai, China) with a heating rate of 20 °C min^−1^ in a nitrogen flow (20 mL min^−1^). XRD (Rigaku Corporation, Osaka, Japan) (Rigaku D/MAX-2400 X-ray diffractometer with Ni-filtered Cu Kα radiation (λ = 1.54056)) was used to investigate the crystal structure of the nanoparticles. Fourier Transform infrared spectroscopy (FTIR) characterization was performed using an infrared spectroscope (PerkinElmer Spectrum 100, Shanghai Perkin Elmer Enterprise Management Co., Ltd., Shanghai, China). Absorption spectra were recorded at room temperature on a Varian Cary 300 Bio UV-vis spectrophotometer (Beijing Purkinje General Instrument Co., Ltd., Beijing, China).

## 3. Results and Discussion

**Figure 1:**
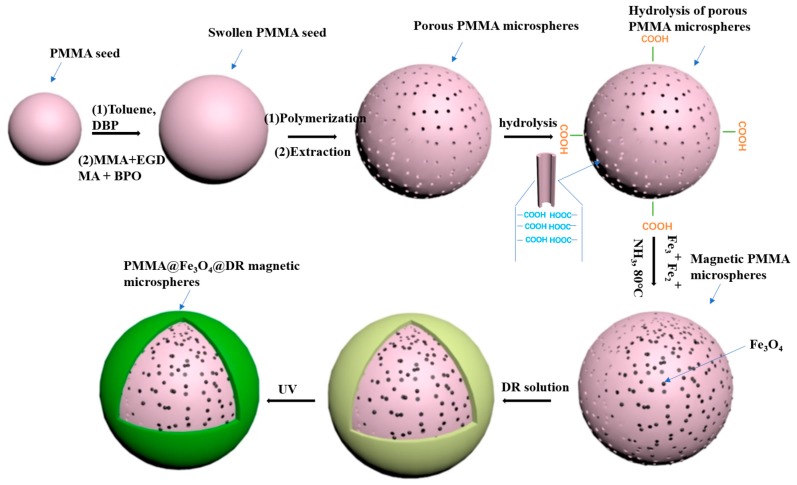
Schematic illustration of the synthesis process used to fabricate (polymethylmethacrylate) PMMA@Fe_3_O_4_@DR magnetic microspheres.

The porous PMMA microspheres were fabricated by seed emulsion polymerization. Figure 1 outlines the steps required for the preparation of PMMA@Fe_3_O_4_@DR. First, monodispersed PMMA seed spheres were prepared by dispersion polymerization. Uniform PMMA seed spheres were swelled in DBP/toluene, followed by adding an emulsion containing monomer MMA, the initiator BPO, and the cross-linker monomer EGDMA. Then, polymerization of the PMMA was carried out within the swollen PMMA seed spheres at 70 °C. The monodispersed porous microspheres were obtained after dissolution of the PMMA seeds and toluene components of the original composite particles. The surface of the porous PMMA microspheres is hydrolyzed to form a negatively charged carboxylate group in NaOH solution. 

**Figure 2:**
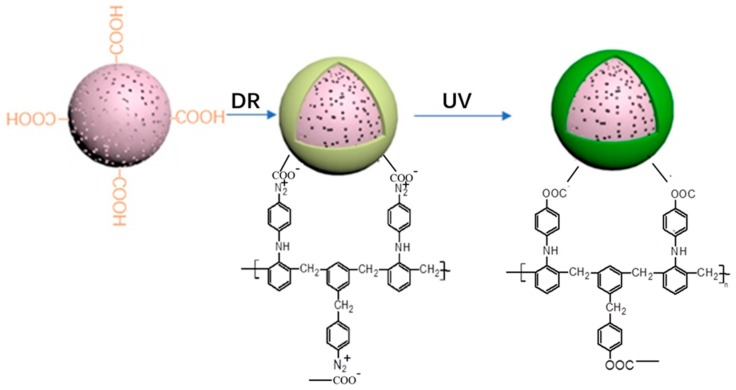
Structural formula of DR and photosensitive reaction process.

Fe^3+^ and Fe^2+^ uptake on the spheres was achieved through ion exchange. Then in-situ formation of Fe_3_O_4_ nanoparticles by ammonia was carried out. Finally, DR was adsorbed onto the surface of the magnetic PMMA microspheres by electrostatic forces, followed by the formation of a stable covalent bond under ultraviolet light irradiation. As a result, the magnetic PMMA microspheres were encapsulated by DR (Figure 2).

**Figure 3:**
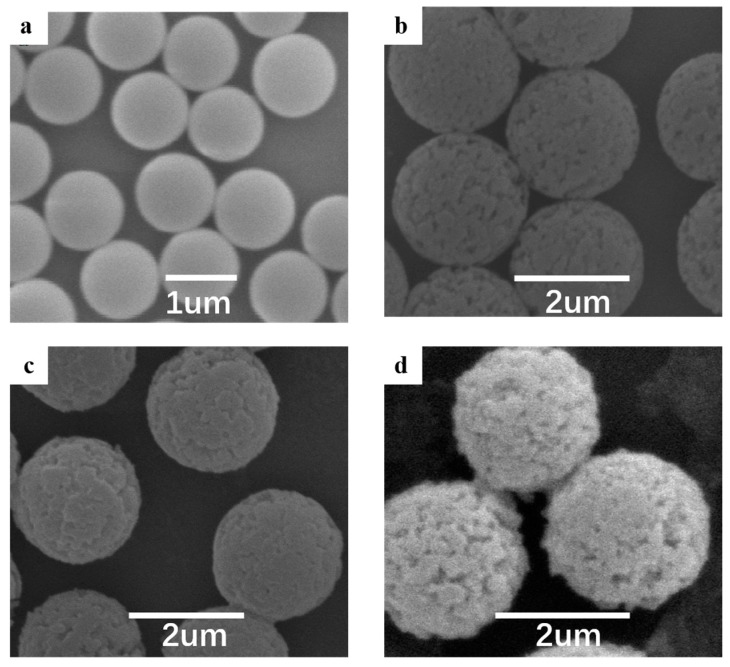
SEM (scanning electron microscopy) images of PMMA seed spheres (**a**); porous PMMA microspheres (**b**); porous magnetic PMMA microspheres (**c**); and DR-encapsulated magnetic PMMA microspheres (**d**).

Figure 3 shows the SEM images of the PMMA seed spheres, porous PMMA microspheres and DR-encapsulated magnetic PMMA microspheres, which demonstrates that they are all perfect spheres with good monodispersity. The sizes of these particles are 1.2 ± 0.1, 2.3 ± 0.1, 2.3 ± 0.1 and 2.4 ± 0.1 µm, respectively. The images clearly show the differences between the surfaces of the porous PMMA microspheres and the original smooth PMMA seed spheres, as well as the differences between the surfaces of the porous PMMA microspheres and the relatively smooth PMMA@Fe_3_O_4_ microspheres. This is because the Fe_3_O_4_ nanoparticles block the pores. Figure 3d shows the more rough PMMA@Fe_3_O_4_@DR microspheres due to DR encapsulation.

**Figure 4:**
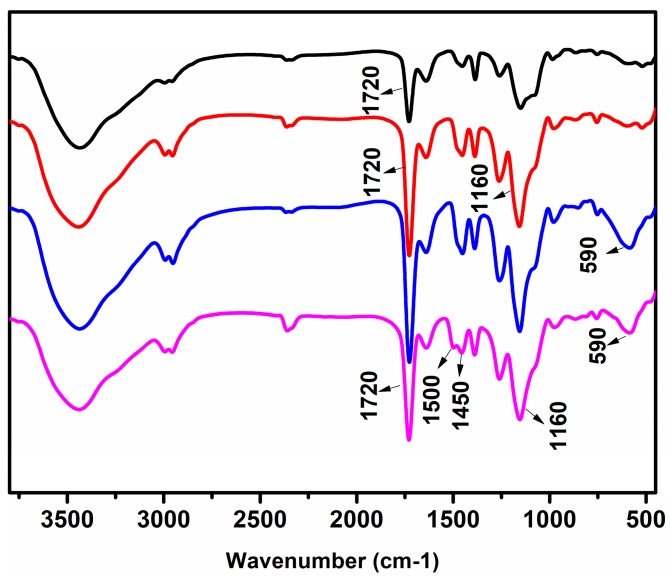
FTIR (Fourier Transform infrared spectroscopy) spectra of (**a**) PMMA; (**b**) PMMA–COOH; (**c**) PMMA@Fe_3_O_4_ and (**d**) PMMA@Fe_3_O_4_@DR microspheres.

FTIR spectra of the porous PMMA, PMMA–COOH, PMMA@Fe_3_O_4_ and PMMA@Fe_3_O_4_@DR microspheres are shown in Figure 4. The spectra in Figure 4 show the characteristic absorption peaks at 3450, 1720, 1500, 1450, 1160, 590 cm^−1^. Spectrum a shows the typical peaks of PMMA microspheres. In spectrum b and c, the –OH peak around 3450 cm^−1^ and –C=O peak around 1720 cm^−1^ significantly increased after hydrolyzation. This indicates that the ester group becomes carboxyl (PMMA–COOH). In spectrum c, the absorption band at 590 cm^−1^ is assigned to the stretching vibration of Fe–O band, indicating that Fe_3_O_4_ nanoparticles are generated inside the porous sphere. In spectrum d, the peaks at around 1450 and 1500 cm^−1^ are indexed to the stretching vibration of the aromatic ring. The peaks at around 1720 cm^−1^ significantly decrease because of hydroxyl groups changing to ester bonds on the surface, which demonstrates that PMMA@Fe_3_O_4_@DR is formed.

**Figure 5:**
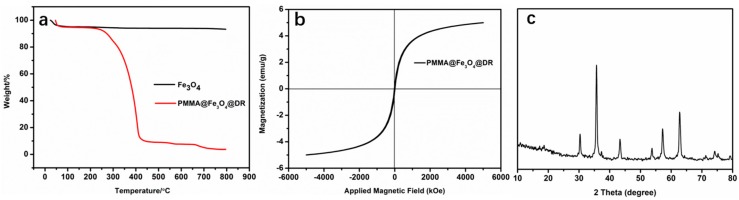
TGA (Thermogravimetric analysis) curves (**a**); magnetization curves (**b**) of magnetic microspheres and XRD (X-ray diffractometer) patterns of Fe_3_O_4_ (**c**).

Thermogravimetric analysis (TGA) curves of PMMA@Fe_3_O_4_@DR and the results are shown in Figure 5a. A tiny mass was detected on the decomposition of Fe_3_O_4_ during the heating process. The major decomposition region of PMMA@Fe_3_O_4_@DR was 250–400 °C, and the organics were almost completely decomposed with the temperature up to 450 °C. The weight fraction of Fe_3_O_4_ in PMMA@Fe_3_O_4_@DR was about 4.9 wt %. The magnetization curves of PMMA@Fe_3_O_4_@DR were obtained by alternate gradient magnetometer (Micro MagTM 2900, Shanghai Yi Hong Scientific Instrument Co., Ltd., Shanghai, China) in the magnetic field ranging from −4.5 to 4.5 kOe at room temperature. The magnetization curves of PMMA@Fe_3_O_4_@DR are depicted in Figure 5b. The magnetization is 4.85 emu g^−1^ in the magnetic field (−4.5 to 4.5 kOe) at room temperature. The XRD patterns of magnetic Fe_3_O_4_ are illustrated in Figure 5c. The five diffraction peaks at 2θ = 30.1°, 35.5°, 45.1°, 57.2° and 62.9° can be observed, which corresponds to (220), (311), (400), (511) and (440) are standard Fe_3_O_4_ crystal. The PMMA@Fe_3_O_4_@DR shows magnetic behavior. Its magnetic properties can be used to facilitate the recovery of adsorbents and dyes. 

**Figure 6:**
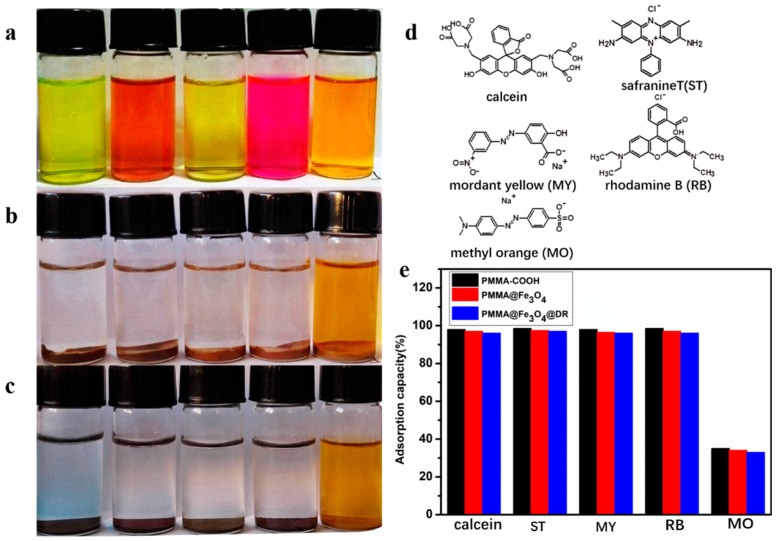
Photographs of aqueous solutions of dyes (**a**) before; (**b**) after adsorption on PMMA@Fe_3_O_4_ adsorbent; and (**c**) after adsorption on PMMA@Fe_3_O_4_@DR adsorbent (from left to right: calcein, ST (safranine T), MY (mordant yellow), RB (rhodamine B), and MO (methyl orange) solution and the initial concentration is 50 mg/L); (**d**) Chemical structures of dyes used for adsorption; (**e**) Saturated adsorption capacities of PMMA-COOH, PMMA@Fe_3_O_4_ and PMMA@Fe_3_O_4_@DR for different dyes.

The purpose of preparing PMMA@Fe_3_O_4_@DR is to obtain a novel adsorbent with excellent adsorption capacity and convenient magnetic separation performance. Various dyes such as rhodamine B (RB), safranine T (ST), mordant yellow (MY), methyl orange (MO) and calcein were chosen to study the adsorption properties of PMMA@Fe_3_O_4_@DR (the molecular structures of five adsorbates are illustrated in Figure 6d). Figure 6a shows an aqueous dye solution, and various bright colors can be clearly observed. As is shown in Figure 6b,c, after the addition of PMMA@Fe_3_O_4_ and PMMA@Fe_3_O_4_@DR respectively, the dye solutions were significantly discolored. In addition, after the adsorption process, the adsorbent can be easily separated by separating the magnets. The different adsorption capacities of the adsorbents were studied in this paper, as shown in Figure 6e. The adsorption capacity of PMMA@Fe_3_O_4_ microspheres was better than that of PMMA@Fe_3_O_4_@DR microspheres, but the difference was very small. We can speculate that the adsorption of PMMA@Fe_3_O_4_ is derived from the strong interaction between the surface –COOH negative groups and the positive functional groups such as –NH_2_, –NH– and =N+H– of the dyes. The RB and ST contain positive =N+H– groups, thus their interactions with PMMA@Fe_3_O_4_ are much stronger than the others. The PMMA@Fe_3_O_4_@DR contains positive –NH– groups, thus it has a strong interaction with the surface –COOH negative groups, such as RB, calcein and AY. PMMA @ Fe_3_O_4_ @ DR has excellent adsorption capacity for ST although it is a cationic dye. This adsorption capacity is due to the electrostatic interaction (unreacted carboxyl group), large surface area and the π–π electron-donor-acceptor interaction of the dye with adsorbent (PMMA @ Fe_3_O_4_ @ DR contains a lot of aromatic rings on the surface). Therefore, our PMMA@Fe_3_O_4_@DR magnetic adsorbent is very suitable for the adsorption of anionic or aromatic dyes.

**Figure 7:**
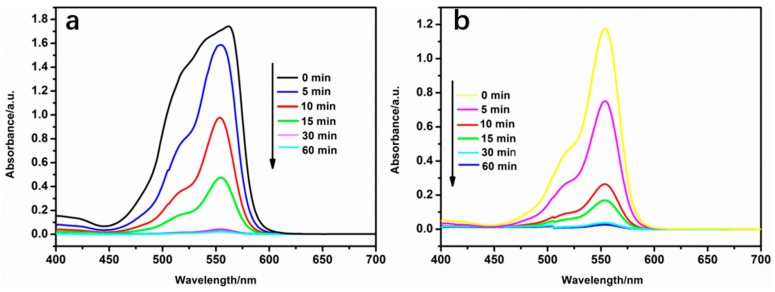
Absorption spectra of (**a**) RB and (**b**) ST at different adsorption times by PMMA@Fe_3_O_4_@DR.

The effect of the contact time on the adsorption of RB and ST dyes by PMMA@Fe_3_O_4_@DR is presented in Figure 7a,b. The initial concentrations of RB and ST were 50 mg/L. The contact time required to achieve equilibrium is an important parameter in practical applications; 99% of the RB and 98% of the ST were adsorbed within 30 mins and both adsorbates reached equilibrium within about 15 min. This is an excellent application for wastewater treatment.

**Figure 8:**
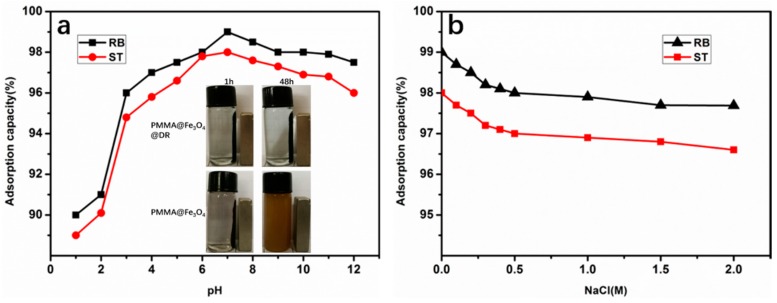
(**a**) Effect of pH on the adsorption of RB and ST by PMMA@Fe_3_O_4_@DR adsorbent at 25 °C and (inset) photographs of PMMA@Fe_3_O_4_@DR and PMMA@Fe_3_O_4_ in pH = 1 for 1 and 48 h; (**b**) Effect of ionic strength on the adsorption of RB and ST by PMMA@Fe_3_O_4_@DR at pH 7 and 25 °C. In both a and b, the initial dyes concentrations of RB and ST are 5 mg/mL.

The effect of pH is another important influencing factor for wastewater treatment. As shown in Figure 8a, there was no significant effect of pH on the dye adsorption of RB and ST from a wide range pH of 4–10. This demonstrates that the electrostatic attraction between the adsorbent and the dye is strong when the pH in the wide range of 4–10. On the other hand, the adsorption capacity decreased significantly at pH 3. This may be attributed to the increase in competition between protons and dyes adsorpting on the PMMA@Fe_3_O_4_@DR surface. Even under the conditions of pH = 1, the PMMA@Fe_3_O_4_@DR still has 90% and 88% of the adsorption capacity of RB and ST, respectively. PMMA@Fe_3_O_4_@DR can maintain good magnetism, while PMMA@Fe_3_O_4_ loses effectiveness in PH= 1 for 48 h (inset Figure 8a). This is very important because industrial waste water typically has a relatively acidic pH range. The effect of the ionic strength on the adsorption is shown in Figure 8b. As the NaCl concentration increases to 2.0 M, the adsorption capacities for RB and ST are not significantly reduced, which demonstrates that PMMA@Fe_3_O_4_@DR can maintain good stability of adsorption under ionic strength.

**Figure 9:**
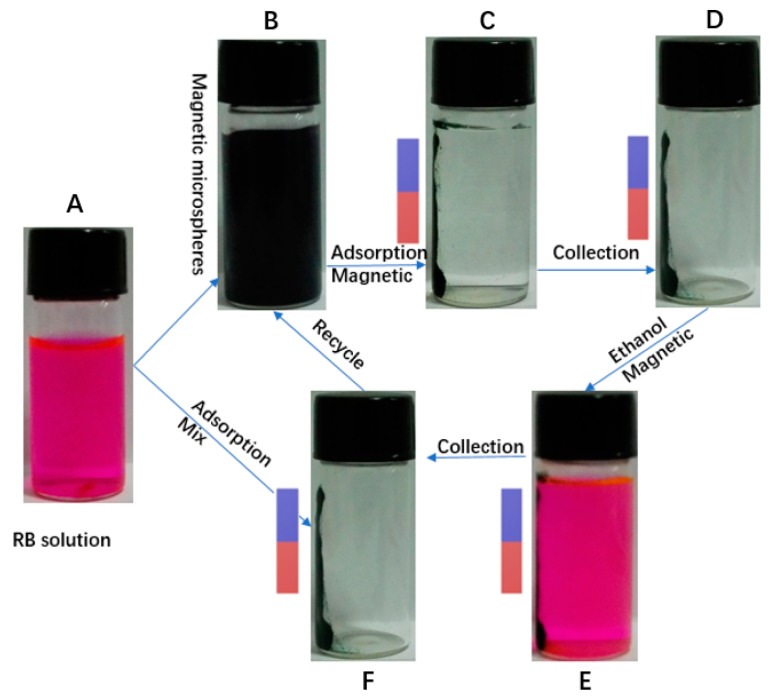
Schematic illustration of magnetic separation of RB from aqueous solution, and regeneration of PMMA@Fe_3_O_4_@DR. RB aqueous solution (**A**); magnetic microspheres and RB solution mixed solution (**B**); magnetic adsorption by magnet (**C**); adsorbent is collected by the magnet (**D**); RB is extracted by ethanol in the magnetic field (**E**); adsorbent is collected by the magnet after extracted (**F**).

PMMA@Fe_3_O_4_@DR (50 mg) was used for removing RB (50 mg/L) from aqueous solution and separated with an external magnetic field. Obviously, the RB solution becomes colorless after magnetic separation (Figure 9), indicating successful adsorption and separation. According to the molecular structure in Figure 6d, there is a negatively charged group (carboxyl group) for RB. Thus, the electrostatic interaction between the carboxyl group and the secondary amine group can be used to adsorb RB, as shown in Figure 9. After extraction with absolute ethanol, the solution returned to its original color (Figure 9e), indicating RB successfully extracted from PMMA@Fe_3_O_4_@DR. The reason is that the electrostatic interaction between the secondary amine group and the carboxylic acid group can be interrupted by an organic solvent such as ethanol. The dye separation and extraction process was repeated three times. Sewage can be purified by recirculation, which is a simple and quick way for recycling. The concentration of RB collected is absorbed by the UV radiation of the standard RB solution. The results showed that 90% RB could be rapidly removed after the first cycle. We believe that this high removal efficiency is due to large surface area and –NH– groups and aromatic rings on the pore.

**Figure 10:**
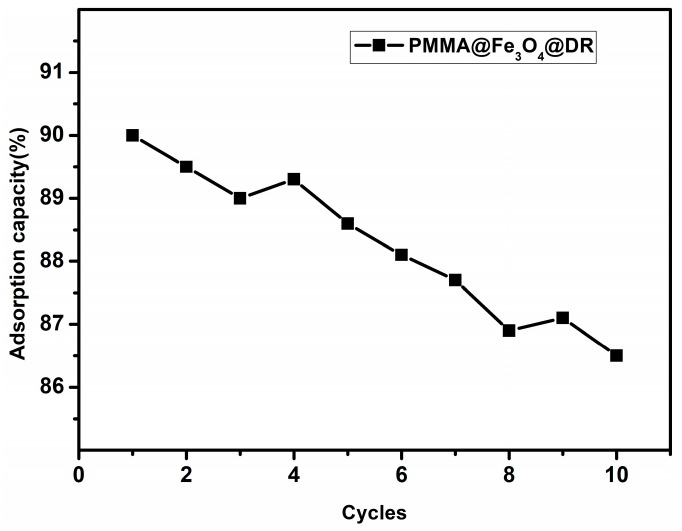
Adsorption ratio of the PMMA@Fe_3_O_4_@DR in 10 cycles of desorption and adsorption compared with the original adsorption capacity.

As shown in Figure 10, more than 86.5% of the adsorption capacity can be retained even after 10 cycles of adsorption. This excellent regeneration performance can be attributed to better solubility and low electrostatic interaction in the ethanol medium. In addition, the dye can be recycled from the ethanol after evaporation and the PMMA@Fe_3_O_4_@DR can be recycled through magnet collection. As a result, our magnetic adsorbents can be easily recycled from industrial water and then reused several times with high adsorption capacity, promising potential in practical applications.

## 4. Conclusions

In summary, we successfully prepared PMMA@Fe_3_O_4_@DR magnetic materials. The photosensitive DR was used to encapsulate the PMMA@Fe_3_O_4_ microspheres to increase the magnetic material stability. The magnetization is 4.85 emu g^−1^ in the magnetic field (−4.5 to 4.5 kOe) and room temperature. We demonstrated that the synthetic PMMA@Fe_3_O_4_@DR can be successfully used as a reusable absorbent for fast and easy removal of anionic and aromatic dyes from wastewater and can maintain excellent magnetic and adsorption properties in harsh environments. Overall, we believe that the approach presented herein provides a convenient way to bind other anionic organic compounds to magnetic absorbents and to quickly separate them from wastewater.
